# Religious and community leaders’ acceptance of rotavirus vaccine introduction in Yogyakarta, Indonesia: a qualitative study

**DOI:** 10.1186/s12889-019-6706-4

**Published:** 2019-04-03

**Authors:** Retna Siwi Padmawati, Anita Heywood, Mei Neni Sitaresmi, Jarir Atthobari, C. Raina MacIntyre, Yati Soenarto, Holly Seale

**Affiliations:** 1 Faculty of Medicine, Universitas Gadjah Mada (UGM), Yogyakarta, Indonesia; 2 School of Public Health and Community Medicine, UNSW Medicine, University of New South Wales, Sydney, Australia

**Keywords:** Rotavirus, Rotavirus vaccine, Immunization, Acceptance, Religious, Islam, Halal, Diarrhea

## Abstract

**Background:**

In Indonesia, oral rotavirus vaccines are available but not funded on the National Immunization Program (NIP). New immunization program introduction requires an assessment of community acceptance. For religiously observant Muslims in Indonesia, vaccine acceptance is further complicated by the use of porcine trypsin during manufacturing and the absence of *halal* labeling. In Indonesia, religious and community leaders and the Majelis Ulama Indonesia (MUI) are important resources for many religiously observant Muslims in decisions regarding the use of medicines, including vaccines. This study aimed to explore the views of religious and community leaders regarding the rotavirus vaccine to inform future communication strategies.

**Methods:**

Twenty semi-structured in-depth interviews were undertaken with religious leaders and community representatives from two districts of Yogyakarta Province, Indonesia. Thematic analysis was undertaken.

**Results:**

Although there was recognition childhood diarrhoea can be severe and a vaccine was needed, few were aware of the vaccine. Participants believed a *halal* label was required for community acceptance, and maintenance of trust in their government and leaders. Participants considered themselves to be key players in promoting the vaccine to the community post-labeling.

**Conclusions:**

This study highlights the need for better stakeholder engagement prior to vaccine availability and the potentially important role of religious and community leaders in rotavirus vaccine acceptability in the majority Muslim community of Yogyakarta, Indonesia. These findings will assist with the development of strategies for new vaccine introduction in Indonesia.

**Electronic supplementary material:**

The online version of this article (10.1186/s12889-019-6706-4) contains supplementary material, which is available to authorized users.

## Background

Rotavirus is the most common cause of severe diarrhea in children less than 5 years old in Indonesia [1, 2]. A 2009 study found 60% of hospitalized and 41% of outpatient children with diarrhea at three major hospitals in Yogyakarta, Indonesia, were rotavirus positive [2]. Since 2011, two rotavirus vaccines, Rotarix® (GSK, Belgium) and RotaTeq® (Merck & Co, USA), have been licensed and are available on the private market in Indonesia. While coverage data is unavailable, vaccine uptake is considered to be low. Despite an economic evaluation indicating the cost-effectiveness of a universal rotavirus immunization program [3]**,** it is not currently included on the government funded National Immunization Program (NIP).

While the vaccine cost on the private market is likely to be prohibitive for the majority of Indonesian children [3], little is known about the other barriers to rotavirus vaccine uptake. Adding complexity to the vaccine decision-making is the public’s acceptance of vaccines which contain animal-derived products and their permissibility under Islamic law. Many vaccines use animal-derived products during the manufacturing process, in the growth culture or use gelatin as a stabilizer. The current oral rotavirus vaccines use porcine trypsin in the manufacturing process. Islam does not have a formalized religious body adjudicating over religious interpretation and faith, nor is there a single Islamic organization for medical sciences that deliberates over the acceptability of products for human consumption, including medicines. Instead, the application of Islamic law includes a continuous discourse between religious scholars (*ulamas*) and laypeople. In 2001, the World Health Organization (WHO) issued a statement on the collective opinion of Islamic religious scholars, pronouncing that animal-derived medical products, including vaccines, that undergo a transformation are considered clean [4]. Despite the WHO statement, not all those of Islamic faith consider vaccines to be *halal* (permissible under Islamic Shariah Law). In situations where the vaccine is still considered *haram* (prohibited), it is permissible with the understanding that it will prevent a life-threating disease and there are no equivalent alternative halal products available. Under this rationale, the use of oral polio vaccine was made permissible by the Majelis Ulama Indonesia (MUI) in 2002 [5]. Acceptance of an oral vaccine may differ from acceptance of parenteral vaccines if there is concern regarding the ingestion of a perceived impermissible substance. While halal certification for the oral rotavirus vaccines have been issued in other countries, a certification has not been issued by MUI.

In Indonesia, registration and approval of medicines, including vaccines, by the Indonesian Bureau of Drug and Food Control (BP POM) does not require a halal certification. The government-funded MUI is Indonesia’s highest Muslim clerical body and has the function of issuing *fatwas* (religious-legal response) including the provision of halal certification. To date, the MUI have issued halal certifications for the two meningococcal vaccines required for the Hajj and a fatwa to permit the use of inactivated polio vaccine (IPV) [5]. For other vaccines, including those on the NIP, no specific halal certifications or fatwas by the MUI exist. For policy-makers, understanding the concerns of MUI members as well as the current debate regarding the introduction of new products in the Islamic community is important to inform policy.

Community acceptance of any new program is contingent on the involvement of key stakeholders in the implementation of a program to suit the community’s needs [6–8]. In Indonesia, religious and community leaders and the MUI are important resources for many religiously observant Muslims in decisions regarding the use of medicines, including vaccines. This study aimed to explore the acceptance by religious and community leaders to the introduction of rotavirus vaccines in Yogyakarta, Indonesia, and their perceptions of the acceptability of the rotavirus vaccine to the communities they represent.

## Methods

### Study design and setting

This qualitative study is a component of a larger study exploring the rotavirus vaccine acceptance among three groups of important decision makers and influencers in community vaccine acceptance [9]: parents, healthcare providers [10] and key community and religious leaders. This paper focuses on community and religious leaders. This study was conducted in the city of Yogyakarta and in the district of Sleman, Yogyakarta Province, Indonesia, representing both urban and rural districts. Community organizations are structured to have branches in the province, district, sub-district and village levels. In Indonesia, Islamic organizations have their own structures following the formation of the formal civil administration at the district and provincial levels. We used the COREQ checklist to report the methods we used in our study (Additional file 1: Table S1: COREQ checklist).

### Participants and recruitment

Participants were purposefully identified. Community leaders were formal sub-district or village leaders and community health workers (volunteer health cadres). Religious leaders were identified at both the sub-district and provincial levels. Specifically, for each sub-district we asked participating parents and healthcare providers to identify cadres, religious leaders, community leaders whom could be approached. Details of participating healthcare workers are reported elsewhere [10] and included purposively selected nurses, midwives, primary care providers and pediatricians from local primary care and hospitals. Parents were pregnant women in their third trimester and primary caregivers of infants aged less than 14 weeks identified by midwives from the registries of community members attending primary health centers in the study area. We included members or leaders of the Islamic religious organizations, Nahdatul Ulama and Muhammadiyah, and the government funded Majelis Ulama Indonesia (MUI) at both the district and provincial level and one member of the Indonesian Bureau of Drug and Food Control (BP POM) in order to explore the rulings on public disclosure of products using porcine in the manufacturing process. Invitation letters were sent to potential respondents and a follow-up telephone call was made to identify interested participants. Respondents were not known to the interviewers prior to interview. Written, informed consent was obtained from all participants at the time of scheduling the interview.

### Respondent characteristics

In all, 20 informants participated in the study, including four religious leaders and seven leaders or staff members of Islamic Organizations. Of the community leaders, four volunteer cadres and four formal community leaders or staff members of sub-district, district, and provincial level offices were interviewed. No observers were present. One member of the central level BP POM was also interviewed. All participants were either high school or college graduates. Prior to the interview, one informant researched rotavirus vaccine on the internet and another obtained information from a healthcare provider. One informant from an Islamic organization was also a practicing pediatrician.

### Data collection

An interview guide, used for interviewers with both community and religious leaders, was developed by the authors to cover themes including knowledge of vaccines on the NIP, perceived severity of diarrhea and the need for a rotavirus vaccine. After initial questioning, an explanation was provided to all participants on rotavirus disease and the rotavirus vaccine including the process of vaccine production. Interviews of 45–60 min duration were conducted in the respondent’s office or home in the local language by experienced researchers (Dr S Padmawati, PhD, Medical Anthropologist and Dr. M Sitaresmi, PhD Pediatrician and public health researcher) from the Faculty of Medicine, Universities Gadjah Mada (UGM) who were familiar with data collection method. Debriefing was done at the end of each interview. Participants were re-contacted if further clarification was required. Findings were discussed weekly by the team and interview guides were modified and revised as needed.

### Analysis

Interviews were audio-recorded, transcribed verbatim, and translated into English. Transcripts were analyzed and manually coded. A code list of major themes was developed through content analysis of the data and were constructed independently by SP and MS, compared and cross-checked and repeated with additional interviews. A final agreed thematic framework was applied to all interviews. Ethics approval was sought and received from the Ethical Review Board of the Faculty of Medicines, UGM and the Human Research Ethics Committee, UNSW Sydney (HC13079).

## Results

### General knowledge and attitudes towards vaccination

Most participants were knowledgeable of the vaccines listed on the current Indonesian NIP, and the consequences of not vaccinating to both the individual and the community.

*“Vaccination is to give protection to children, in Islam it is permitted…because it has “kemaslahatan” or for the collective good” (Religious leader, 47 years).*

*“If not vaccinated, the children’s endurance to many diseases is lacking so that they could be easily infected with the diseases. For instance, measles easily infects others, even if only one [child] who was not immunized has measles, other [children] would get the same disease” (Cadre, 53 years).*

### Mild disease unless untreated

There was an overarching belief that mild diarrhea was common in children under five and very easy to treat. Some participants associated diarrhea as a normal sign of child development. However, it was acknowledged that mild diarrhea could progress to severe diarrhea if the child became dehydrated. Furthermore, it was suggested that while severe diarrhea is very rare, children under five are vulnerable and late management could be fatal. All participants felt that personal and environmental hygiene, especially proper hand washing, maintaining a good diet and healthy living could prevent diarrhea. Only four participants said a virus could cause diarrhea and only three mentioned (unprompted) that it could be prevented by vaccination.

*“... the diseases are sourced from the hand. So I recommend that mothers with young children wash their hands when they want to eat or feed their young children, wash the hands well with soap because if not [with soap] it’s useless ... hand-washing with soap is not the custom of the people here...”* (Cadre, 42 years old).

### Low underlying knowledge about rotavirus disease

The majority of participants had never heard of rotavirus as a cause of diarrhea, except three participants (a health provider and member of an Islamic organization; a local health cadre; and a member of an Islamic organization who reported researching the topic prior to the interview). When the symptoms of rotavirus diarrhea were described to participants, most recognized it as “*muntaber*” or *muntah berak* (vomiting and defecating*)* in local terms and described it as very severe and serious. However, few had observed such cases and none reported personal experience with “*muntaber*” or hospitalization for diarrheal disease amongst family members.

### Knowledge and attitudes towards rotavirus vaccination

Only three respondents were aware of the rotavirus vaccine. After receiving information on the rotavirus vaccine, all participants perceived the vaccine as being important for children in their community and anticipated community acceptance of a vaccine preventing diarrhea. However, all agreed that in the absence of NIP funding, the current cost of the vaccine on the private market (cited by participants as around 200,000 rupiah (Rp) (US$18) for each dose, actual cost 210,000–280,000 Rp) was beyond the reach of most parents. Participant felt that given the majority of rotavirus cases are very mild, the benefit of the vaccines was not worth the cost to parents. Many informants estimated that people could afford a vaccine which was priced around 50,000 Rp (US$4) per dose. One informant specifically indicated that the vaccine was a good investment as diarrhea could be fatal, and the cost was favorable compared to the pentavalent diphtheria-tetanus-pertussis (DTP), hepatitis B, *Haemophilius influenzae* type b vaccine, also available on the private market in Indonesia at a cost of 400,000 Rp (US$32) per dose.

Almost all community and religious leaders raised the importance of including rotavirus vaccine into the national program and the right of the parent to refuse non-compulsory vaccines. By including the vaccine on the NIP, it would communicate to the community the message that the disease is severe especially for children, and the government is committed to the prevention of the disease. In the absence of NIP listing, they also suggested the price of the vaccine should be relatively cheap or provided under national (Jamkesmas) or local (Jamkesda) health insurance, to be advantageous to the community.

“*…using and not using the vaccine is an individual decision, unlike the fogging for dengue which is compulsory and [refusal of insecticide fogging] would harm the community, vaccine for diarrhea is an individual right” (leader of an Islamic Organization, 63 years).*

### Disclosure of porcine content and the need for a “fatwa”

Participants perceived the community would be divided in their decision to accept or reject the vaccine due to the association with porcine.

*“…One group of people will see that porcine or anything related to pig/pork is prohibited because it is haram in Islam. Thus, people will refuse to consume the product. But one other group will see the needs over the product. If many lives will be at stake because we do not use the product, then, the product will be permitted to use. However, endorsement from the ulamas is needed so that people will have no doubt to use it...” (Religious leader, 53 years).*

According to the religious leaders, such decisions for fatwa could be decided with *ijtihad* (independent reasoning) based on the agreement by all ulamas about the product. Participants described the need for policy makers to work with ulamas to provide rulings that the product is halal and that different interpretations were likely. Religious leaders predicted the vaccine being interpreted as haram but still permitted for use because of the potential for saving children’s lives in the absence of other equivalent halal products. It was suggested that the ‘state of emergency’ ruling for the use of a vaccine could not be used all the time, and there should be effort given to the development of halal vaccines.

Both religious and community leaders considered whether specifically informing the community of the use of porcine during the vaccine manufacturing was necessary if there was a MUI halal certification, indicating disclosure could fragment the community in their opinions. However, the BP POM participant confirmed it is a regulatory requirement to clearly state if products have direct or indirect contact with porcine derived products in the production process.

*“…for the lay people, we don’t need to explain about the process of vaccine development which uses porcine; the important thing is the label of ‘halal’ by MUI. The key is that the leaders have processed it and it is considered ‘halal’…” (Community leader, 52 years).*

*“…no need to tell about the process [of vaccine development]. If there was a ‘halal’ label most of the people would accept that. The sin and the risk [of committing a sin] will be the responsibility of the vaccine manufacturers.” (Religious leader, 48 years).*

### The role of religious and community leaders in endorsing the use of vaccines

It was suggested that religious and community leaders could play two important roles. The first is prior to vaccine endorsement by the MUI, and the second is after a fatwa is announced. Religious leaders felt they could assist with providing evidence to the development of a fatwa and in advocating to the MUI to endorse the vaccine. After a fatwa had been announced, community and religious leaders described their role in announcing the fatwa during congregation meetings, and in discussions with other religious and community leaders. However, they acknowledged a need for information from the health authority or healthcare providers to assist them in the promotion of the vaccine. Religious and community leaders considered the importance of a communication plan including tools to inform and educate them, including information about the manufacturing process along with assurances that the final product has been “washed” more than seven times and that no trace of the porcine remains in the product as well as information on the burden of rotavirus diarrhea.

*“…So the point is this... Ulama will say that there are so many diseases, if this is not treated, it will more dangerous [for children] ... that’s how the fatwa is recommended…. otherwise [the fatwa] will not be developed….” (Religious leader, 52 years).*

*“…There is a need for religious and community leaders to talk to the people. They will use [the vaccine] if it is free….they do not want to pay a lot of money….they thought it is only diarrhea anyway….” (Community leader, 46 years).*

“*So people have closed their hearts not to accept [vaccines] ... thus, [the strategy] for people like this must be approaching the leaders ... because they are people who obey the leaders and usually do not want to listen to outsiders.” (Religious leader, 52 years).*

## Discussion

Amongst the religious and community leaders interviewed there was unanimous agreement - in order to successful promote the rotavirus vaccine in Indonesia it must be appropriately labeled as halal and included in the government program. Religious and community leaders are willing to provide support in the introduction of new vaccines and felt their role is not limited to the post-introduction period. Their role should include advocacy for immunization prior to the introduction of a new vaccine, during implementation, and as part of the ongoing program [7].

Based on their prior experience, participants believed either a halal certification or a fatwa from the MUI regarding the permissibility of the vaccine would have a significant impact on community acceptance. This consensus is in line with recommendations from a 2011 workshop discussing the rotavirus vaccine conducted by the Faculty of Medicine, UGM (unpublished) which included representatives from MUI, other Islamic organizations, the BP OM and the Indonesian Pediatric Association. The impact associated with a lack of religious endorsement has already been established for other vaccines. For example, the non-halal status was reported as a barrier by mothers in a study of pneumococcal vaccine uptake in Bandung, Indonesia [11] and was an important barrier to the acceptance of the influenza A H1N1 vaccine for Muslim Malays [12].

Assurance that the vaccine is halal is a complex issue for many religiously observant Muslims and knowledge of the religious aspects of parental decision-making is important for healthcare providers [13] and health authorities. Given their influence in the community, religious leaders play an important role in providing the bridge between immunization programs and the community, particularly during the introduction of a new vaccine [7]. This view was reiterated by healthcare providers who considered strong positioning by religious leaders, including the issuing of a halal label by the MUI were key to a high vaccine acceptance by parents [10]. While current NIP vaccines achieve high coverage in Indonesia (despite some not having halal certification), community and religious leader support may be especially important for new vaccines such as the rotavirus vaccine. Stakeholder engagement in establishing social norms plays a key role in many parents decision to vaccinate their children [14, 15] and as such they should be equipped with adequate information [8] and engaged at different stages of new vaccine rollout. At a global level, the WHO SAGE Vaccine Hesitancy Working Group acknowledge the influence of community leaders, including religious leaders on vaccine acceptance the positive effects of interventions that engage religious or other influential leaders to promote vaccination in the community [16, 17].

Interviewing the community and religious leaders is an important part of public engagement, although for this study, it was limited to local religious and community leaders in one province. The use of in-depth interviews to elicit a greater depth in the information is a key strength of our work. However, we acknowledge interviews were only undertaken with selected group of participants, so the possibility of other important themes emerging cannot be ruled out. The MUI is a national body, and therefore their rulings encompass all of Indonesia and these recommendations of participants in our study may be generalized to other areas of Indonesia. However, generalizability may be limited in that not all Islamic parents depend on the recommendations of religious leaders for making decisions related to vaccination. In addition, information was not captured regarding the reasons why participants declined to participate. Despite this, the findings may inform national or community-wide communication strategies.

## Conclusions

Community and religious leaders in our study were broadly accepting of the rotavirus vaccine. The permissibility of the vaccine under Islamic law and the high cost on the private market were seen as significant barriers to vaccine uptake, in the absence of a national program. This study highlights the need to engage community and religious leaders at all levels, including provision of adequate information on vaccines prior to availability in the community.

## Additional file

Additional file 1: COREQ checklist. (DOCX 18 kb)

## Abbreviation

BP POM: Indonesian Bureau of Drug and Food Control
DTP: Diphtheria-tetanus-pertussis vaccine
GSK: GlaxoSmithKline
IPV: Inactivated polio vaccine
MUI: Majelis Ulama Indonesia
NIP: National Immunization Program
Rp: Rupiah
UGM: Universities Gadjah Mada, Yogyakarta, Indonesia
UNSW: University of New South Wales, Sydney, Australia
WHO: World Health Organization
